# Development of in-house serological methods for diagnosis and surveillance of chikungunya

**DOI:** 10.26633/RPSP.2017.56

**Published:** 2017-06-29

**Authors:** Saira Saborío Galo, Karla González, Yolanda Téllez, Nadezna García, Leonel Pérez, Lionel Gresh, Eva Harris, Ángel Balmaseda

**Affiliations:** 1 National Virology Laboratory, Centro Nacional de Diagnóstico y Referencia Ministry of Health Managua Nicaragua National Virology Laboratory, Centro Nacional de Diagnóstico y Referencia, Ministry of Health, Managua, Nicaragua.; 2 Sustainable Sciences Institute Sustainable Sciences Institute Managua Nicaragua Sustainable Sciences Institute, Managua, Nicaragua.; 3 Division of Infectious Diseases and Vaccinology, School of Public Health University of California BerkeleyCalifornia United States of America Division of Infectious Diseases and Vaccinology, School of Public Health, University of California, Berkeley, California, United States of America.

**Keywords:** Chikungunya virus, clinical laboratory techniques, enzyme-linked immunosorbent assay, Nicaragua, Central America, Virus chikungunya, técnicas de laboratorio clínico, ensayo de inmunoadsorción enzimática, Nicaragua, América Central

## Abstract

**Objective.:**

To develop and evaluate serological methods for chikungunya diagnosis and research in Nicaragua.

**Methods.:**

Two IgM ELISA capture systems (MAC-ELISA) for diagnosis of acute chikungunya virus (CHIKV) infections, and two Inhibition ELISA Methods (IEM) to measure total antibodies against CHIKV were developed using monoclonal antibodies (mAbs) and hyperimmune serum at the National Virology Laboratory of Nicaragua in 2014–2015. The sensitivity, specificity, predictive values, and agreement of the MAC-ELISAs were obtained by comparing the results of 198 samples (116 positive; 82 negative) with the Centers for Disease Control and Prevention’s IgM ELISA (Atlanta, Georgia, United States; CDC-MAC-ELISA). For clinical evaluation of the four serological techniques, 260 paired acute and convalescent phase serum samples of suspected chikungunya cases were used.

**Results.:**

All four assays were standardized by determining the optimal concentrations of the different reagents. Processing times were substantially reduced compared to the CDC-MAC-ELISA. For the MAC-ELISA systems, a sensitivity of 96.6% and 97.4%, and a specificity of 98.8% and 91.5% were obtained using mAb and hyperimmune serum, respectively, compared with the CDC method. Clinical evaluation of the four serological techniques versus the CDC real-time RT-PCR assay resulted in a sensitivity of 95.7% and a specificity of 88.8%–95.9%.

**Conclusion.:**

Two MAC-ELISA and two IEM systems were standardized, demonstrating very good quality for chikungunya diagnosis and research demands. This will achieve more efficient epidemiological surveillance in Nicaragua, the first country in Central America to produce its own reagents for serological diagnosis of CHIKV. The methods evaluated here can be applied in other countries and will contribute to sustainable diagnostic systems to combat the disease.

## RESUMEN

**Objetivo.:**

Elaborar y evaluar métodos serológicos para el diagnóstico y la investigación del chikungunya.

**Métodos.:**

Se elaboraron dos sistemas de ELISA de captura de IgM (MAC-ELISA por sus siglas en inglés) para el diagnóstico de la infección aguda por el virus de (CHIKV) y dos métodos de ELISA de inhibición (MEI) para determinar el valor cuantitativo de los anticuerpos totales contra el CHIKV, en el Laboratorio Nacional de Virología de Nicaragua en 2014–2015, para lo cual se utilizaron anticuerpos monoclonales (AcMo) y sueros hiperinmunes. Se determinó la sensibilidad, la especificidad y los valores predictivos, así como la concordancia de los MAC-ELISA, comparando los resultados de 198 muestras (116 positivas y 82 negativas) con el ELISA de los Centros para el Control y la Prevención de Enfermedades de los Estados Unidos (Atlanta; MACELISA-CDC). Para la evaluación clínica de las cuatro técnicas serológicas, se emplearon 260 muestras de suero obtenidas en la fase aguda y en la fase de convalecencia de presuntos casos de chikungunya.

**Resultados.:**

Se estandarizaron los cuatro métodos analíticos determinando las concentraciones óptimas de los diferentes reactivos. La duración del procesamiento se redujo sustancialmente en comparación con el MAC-ELISA-CDC. Con los sistemas de MAC-ELISA, se obtuvo una sensibilidad del 96,6% y del 97,4% y una especificidad del 98,8% y del 91,5% al utilizar AcMo y suero hiperinmune, respectivamente, en comparación con el método de los CDC. La evaluación clínica de las cuatro técnicas serológicas, en comparación con la PCR en tiempo real de los CDC, arrojó una sensibilidad del 95,7% y una especificidad del 88,8%–95,9%.

**Conclusiones.:**

Se estandarizaron dos sistemas de ELISA-MAC y dos de MEI y se comprobó que poseen la calidad adecuada para el diagnóstico y las investigaciones del chikungunya, con lo cual mejorará la eficiencia de la vigilancia epidemiológica en Nicaragua, el primer país centroamericano que produce sus propios reactivos para el diagnóstico serológico del CHIKV. Los métodos estudiados en este trabajo pueden aplicarse en otros países y contribuyen al desarrollo de sistemas de diagnóstico sostenibles para combatir la enfermedad.

Chikungunya (CHIKV) is a reemerging arbovirus that belongs to the *Alphavirus* genus of the *Togaviridae* family. The 60 nm – 70 nm diameter virion has a phospholipid envelope and a singlestranded positive-sense ribonucleic acid (RNA) genome that encodes three structural and seven nonstructural proteins (1, 2). It has been divided into three phylogenetic genotypes based on the gene sequences of Envelope protein E1: Asian, East/Central/South African, and West African (3). CHIKV is transmitted to humans by the bite of an infected *Aedes aegypti* or *Aedes albopictus* mosquito (1). The disease is characterized by acute onset of fever accompanied by arthralgia, myalgia, and headache; skin rash may occur in 40% – 50% of cases (1 – 3). Chikungunya is endemic in Africa and Southeast Asia, where most epidemics occurred in the 1960s and 1990s (2). From 2005 – 2007, an estimated 1.3 million people were reported to be infected in India; CHIKV was also widespread in Malaysia, Sri Lanka, Indonesia, and the island of La Reunion (2 – 7).

In December 2013, the Pan American Health Organization (PAHO) reported the first case of CHIKV transmission in the Americas. The virus quickly spread from St. Martin to other countries in the Caribbean, as well as to Central and South America (8 – 10). Through May 2015, approximately 1.4 million people with suspected chikungunya were reported in the Americas, with an incidence rate of 146.3 per 100 000 inhabitants (11). In Nicaragua, the first autochthonous case of chikungunya was reported in September 2014; through May 2015, a total of 4 153 confirmed cases and 19 544 suspected cases were reported (11).

In Nicaragua, epidemiological surveillance of chikungunya was based on Immunoglobulin M (IgM) detection in suspected cases after the 4th day of illness (8). Initially, the IgM capture Enzyme-Linked Immunosorbent Assay (MAC-ELISA) developed by the Centers for Disease Control and Prevention (CDC, Atlanta, United States) was used for serological diagnosis of chikungunya (8). Demand was particularly high in Nicaragua because its national model for epidemiological surveillance is based on active case-finding in the community. Limited donations of reagents by the CDC and the prohibitive cost of commercial kits made developing in-house techniques a priority. The study authors were able to rapidly obtain a chikungunya vaccine strain (13, 14) and anti-CHIKV monoclonal antibodies (mAbs; 12) from colleagues at Washington University (St. Louis, Missouri, United States). Using these reagents, the authors developed a MAC-ELISA for the diagnosis and surveillance of chikungunya.

Additionally, a goal was set to implement a surveillance system similar to the one in place for dengue surveillance, where most of the reagents for dengue-specific IgM antibody detection are produced at the National Virology Laboratory (Managua, Nicaragua) and distributed to 12 laboratories throughout the country. Therefore, the researchers produced a rabbit hyperimmune serum in order to establish a sustainable chikungunya diagnostic and surveillance system through the development of an additional MAC-ELISA. Furthermore, to help predict epidemiological behavior in the coming years, it was necessary to perform focal and national seroprevalence studies to determine the extent of the current epidemic.

To this end, two Inhibition ELISA methods (IEM), using the mAbs or hyperimmune serum, were developed to determine total anti-CHIKV antibodies. The IEMs were based on the methods described by Vazquez and Fernandez for dengue (15, 16). All methods showed high sensitivity, specificity, and predictive values, sufficient to achieve the goals of chikungunya diagnosis and surveillance, and they were implemented immediately in the Nicaraguan Ministry of Health.

## MATERIALS AND METHODS

### Clinical samples

To determine the cutoff (or positivity threshold) values of both MAC-ELISA methods, 137 samples (92 negative and 45 positive, as determined using the CDC-MAC-ELISA) were processed. The sensitivity, specificity, predictive values, and agreement of both MAC-ELISA systems in comparison with the CDC-MAC-ELISA were determined using 198 additional samples (116 positive and 82 negative), collected an average of 5.9 days after symptom onset (interquartile range [IQR]: 1). All samples consisted of sera from suspected chikungunya cases collected through the national surveillance programs of Nicaragua. The detailed protocol of the CDC-MAC-ELISA is described elsewhere (8). For the clinical evaluation of the four serological methods, the analysis used 260 paired acute and convalescent sera—on average collected 2.0 days (IQR: 1) and 21.6 days (IQR: 8), respectively, after symptom onset from patients in two ongoing prospective studies of dengue in Nicaragua (17, 18).

These studies were approved by the Institutional Review Boards of the Centro Nacional de Diagnóstico y Referencia (CNDR) of the Ministry of Health of Nicaragua and the University of California–Berkeley (Berkeley, California, United States). The reference method for the clinical evaluation was the CHIKV real-time Reverse-Transcription-Polymerase Chain Reaction (rRT-PCR) recommended by the CDC.

### Production of rabbit hyperimmune serum

In order to obtain hyperimmune serum (HIS), the vaccine strain CHIKV 181/25 (13, 14) was propagated in Vero cells. Previously vitaminized and dewormed 4-month-old white New Zealand rabbits were inoculated according to the following immunization schedule: 1 ml of virus concentrated by osmosis using refined sugar via intravenous route and 8 ml of supernatant from Vero cells infected with CHIKV via intramuscular route in two different sites on days 0, 15, 30, and 37. On day 42, a blood sample was drawn. Anti-CHIKV antibodies from the serum were titrated using IEM and compared with a non-immune serum sample drawn prior to initiation of the immunization schedule. Once the high titers were confirmed, an intrathoracic puncture was performed to collect blood and obtain the HIS. Serum was aliquoted and stored at -80° C until further use (19).

### Conjugation of anti-CHIKV hyperimmune serum and monoclonal antibody

Anti-CHIKV gamma globulin was precipitated from the HIS using saturated ammonium sulfate, and then conjugated to horseradish peroxidase (HRP) using the periodate oxidation method (20). An anti-CHIK mAb (CHIKV 152) generated by at Washington University (St. Louis, Missouri, United States) by Michael S. Diamond (12) was conjugated to HRP using the kit EZ-Link plus activated peroxidase (Thermo Fisher Scientific Inc., Waltham, Maryland, United States). The titers of HIS (41 000) and CHIKV 152 mAb (35 000) were determined by IEM.

### CHIKV antigen production

CHIKV antigen was obtained by inoculating the vaccine strain CHIKV 181/25 (13, 14) into *Aedes albopictus* C6/36 HT cells at 33°C in 175-cm2 flasks for 5 days under Biosafety Level (BSL) 2 conditions since 181/25 is a vaccine strain. Three freeze-thaw cycles were performed, then the flask content was centrifuged at 3 500 rpm for 30 minutes (min), and the supernatant was concentrated by osmosis with refined sugar for 24 hours (hr). The presence of the virus was confirmed by rRT-PCR. The antigen was inactivated with formaldehyde (1 : 2 000) and stored at –80° C until further use.

### IgM capture ELISA using hyperimmune serum and monoclonal antibody

Strips of polystyrene microwells (flat-bottom Nunc® Immuno MaxiSorp, Sigma-Aldrich Corporation, St. Louis, Missouri, United States) were coated overnight at room temperature with 100 µl per well of anti-human IgM immunoglobulin at a concentration of 5 µg/ml and 2.5 µg/ml in carbonate buffer (0.05 M, pH 9.6) for the IgM capture ELISA using hyperimmune serum (MAC-ELISA-HIS) and monoclonal antibody (MAC-ELISA-mAb), respectively. Wells were washed three times with phosphate-buffered saline (PBS) containing 0.05% Tween20 (PBS-T) and blocked with 1% bovine serum albumin (BSA) in PBS-T for 30 min at 37°C. Then 50 µl of serum diluted 1 : 20 in PBS-T was added and incubated for 1 hour at 37°C. After four washes with PBS-T, 50 µl of CHIKV antigen (1:25 dilution for MAC-ELISA-HIS and 1:50 for the MAC-ELISA-mAb) was added and incubated for 1 hr at 37°C, then washed four times with PBS-T. HRP-conjugated anti-CHIKV antibodies were then added as follows: for the MAC-ELISA-HIS, 50 µl of a 1 : 10 000 dilution of HRP-conjugated anti-CHIKV HIS for 30 min at 37°C; and for the MAC-ELISA-mAb, 50 µl of a 1:10 000 dilution of HRP-conjugated CHIKV mAb 152 for 1 h at 37°C. After five washes with PBS-T, 50 µl of substrate tetramethylbenzidine (Calbiochem) was added for 10 min at room temperature, and the reaction was terminated by the addition of 50 µl of 12.5% sulfuric acid. The optical density (OD) at 450 nm was measured in an ELISA reader (Thermo Electron Corporation, Shanghai, China). Samples with an OD higher than the average of the negative controls plus 3 standard deviations for MAC-ELISA-HIS and greater than the average of the negative controls plus 4 standard deviations for MAC-ELISA- mAb were considered positive. Positive and negative controls consisted of serum samples with known anti-CHIKV IgM responses, as determined using the CDC-MAC-ELISA, that tested negative for Hepatitis B, C, and human immunodeficiency virus (HIV).

### Inhibition ELISA methods using hyperimmune serum and monoclonal antibody

In all, 96 well-polystyrene plates (Nunc Immuno MaxiSorp) were coated overnight at room temperature with 100 µl per well of 10 µg/ml anti-CHIKV HIS (IEM-HIS) or with 5 µg/ml of CHIKV mAb 187 (IEM-mAb; 12) in carbonate buffer. The plates were washed three times with PBS-T and blocked with 1% BSA in PBS-T for 30 min at 37°C. Then, 100 µl of antigen (1 : 100 dilution for both IEM) was added for 1 hr at 37°C. After four washes with PBS-T, 100 µl of serum (serially diluted in PBS-T with 0.5% BSA from 1:10 to 1 : 100 000), positive control (1:5 120 dilution), and negative control (PBS-T) was added. The plates were then incubated for 2 hr at 37°C. After four washes with PBS-T, 100 µl of HRP-conjugated anti-CHIKV HIS or mAb 152 diluted 1:10 000 in PBS-T with 2.5% of normal bovine serum was added. The plates were incubated for 1 hr at 37°C and washed five times with PBS-T. The HRP-catalyzed reaction was carried out as described for the MAC-ELISA methods. The percentage inhibition (%I) was calculated using the following formula: %I = (1-[sample OD/mean OD of negative controls]) x 100%. Sample titer was then calculated using the Reed and Muench method (21), according to the following formula:

Titer = exponential [(%I of the lowest sample dilution with a %I ? 50% inhibition - %I of the highest sample dilution with a %I < 50%) / (%I of the lowest sample dilution with a %≥ 50% inhibition – 50) + log of the lowest sample dilution with a %I ? 50% inhibition]

### Statistical analysis

Sample size was calculated using Epidat® software version 4.1 (Consellería de Sanidade, Xunta de Galicia, Santiago de Compostela, Galicia, Spain; PAHO; Universidad CES, Medellín, Antioquia, Colombia) and the following parameters: precision of the sensitivity and specificity estimates, 5%; expected sensitivity and specificity, 95%; and confidence level, 95%. The calculated sample size was at least 193 samples. The sensitivity, specificity, positive and negative predictive values, and their 95% Confidence Intervals (95% CI), as well as kappa indices, were calculated using the statistical package Stata®/MP13.1 (StataCorp LP, College Station, Texas, United States).

## RESULTS

### Development of the CHIKV MAC-ELISA and Inhibition ELISA methods

**FIGURE 1:**
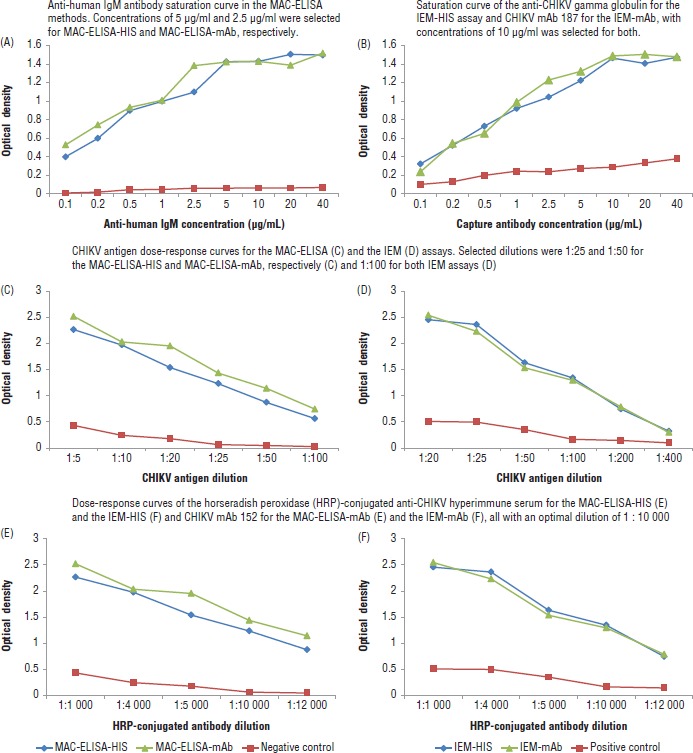
Determination of the optimal concentrations of key components of the chikungunya virus (CHIKV) Enzyme-Linked Immunosorbent Assay (ELISA) methods

First, the optimal concentrations of the major components of the four serological tests were established. Optimal concentrations of MAC-ELISA-HIS and MAC-ELISA-mAb were determined using a saturation curve (Figure 1A). Optimal concentrations were 5 µg/ml and 2.5 µg/ml for MAC-ELISA-HIS and MAC-ELISA-mAb, respectively, as no significant increase in the OD of the positive controls was detected at higher concentrations. Similarly, the optimal capture antibody concentration was determined for both IEMs. The optimal concentration of both IEM-HIS and CHIKV 187 mAb was 10 µg/ml (Figure 1B). The optimal concentrations of CHIKV antigen and HRP-conjugated anti-CHIKV antibody were selected as those yielding the best discrimination between the positive and negative controls (Figure 1C – 1F). Moreover, for the MAC-ELISA methods, the selected dilutions generated OD values > 1 and < 0.1 for positive and negative controls, respectively. For the IEM, the selected dilutions had to result in OD > 1 for the negative control. Using these criteria, the optimal dilutions for the MAC-ELISA-HIS were 1:25 and 1:10 000 for the antigen and the HRP-conjugated anti-CHIKV antibody, respectively (Figure 1C and 1E). For the MAC-ELISA-mAb, the antigen was optimal at a dilution of 1 : 50, and the anti-CHIKV antibody, at a dilution of 1 : 10 000 (Figure 1C and 1E). For both IEMs, Figures 1D and 1F show the optimal dilutions for IEM, which were 1 : 100 for the antigen and 1 : 10 000 for the anti-CHIKV antibody.

### In-house MAC-ELISA versus CDC-MAC-ELISA

A summary of the protocols for the two newly developed, in-house MAC-ELISA systems compared to the CDC-MAC-ELISA (8) is shown in Table 1. A substantial reduction in processing time was achieved for both in-house MAC-ELISA systems as compared to the CDC method. Taking into account that the plates can be coated with anti-human IgM antibody, blocked, and stored until further use, the CDC method takes 2 days, while results from the in-house MAC-ELISA were obtained in approximately 3 hours, varying slightly depending on the number of samples processed.

A total of 198 serum samples collected from suspected chikungunya cases with known CDC-MAC-ELISA results were processed using the two in-house MAC- ELISA systems and compared to the CDC method. Both in-house systems yielded sensitivity, specificity, and predictive values above 90% (Table 2) when using the CDC-MAC-ELISA as a reference. Moreover, very good agreement with the CDC-MAC-ELISA was obtained, as indicated by the kappa index (Table 2). All discordant samples as compared to the CDC-MAC-ELISA, namely four (1 false-positive and 3 false-negatives) by MAC-ELISA-HIS and 11 (7 false-positives and 4 false-negatives) by MAC-ELISA-mAb, had OD values very close to the cut-off value.

**TABLE 1.** Comparison of the in-house and the Centers for Disease Control and Prevention (CDC) MAC-ELISAs, Nicaragua, 2014-2015

Steps and reagents	CDC	MAC-ELISA His^a^	mAb^b^
Anti-human IgM	2.5 pg/ml	5 pg/ml	2.5 pg/ml
Blocking solution	5% milk	1% BSA^c^	1% BSA
Sample dilution	1 : 400	1 : 20	1 : 20
Viral antigen	1 : 320	1 : 25	1 : 50
Non-viral antigen	yes	NA^d^	NA
Incubation time	16-18 hours	1 hour	1 hour
HRP^e^-conjugated anti-CHIKV antibody	1 : 4 000	1 : 10 000	1 : 10 000
Number of washes	10 times	5 times	5 times
Substrate (TMB^f^)	75 pl	50 pl	50 pl
Samples per plate	45	90	90
Total processing time from addition of the sample	48 hours	2.5 hours	3 hours

a Hyperimmune serum.

b Monoclonal antibody 152.

c Bovine serum albumin.

d Not applicable.

e Horseradish peroxidase.

f Tetramethylbenzidine.

**TABLE 2.** Performance of the in-house MAC-ELISA systems compared to the Centers for Disease Control and Prevention MAC-ELISA (*n*=198), Nicaragua, 2014-2015

Methods	Sensitivity (95% CI)	Specificity (95% CI)	Positive predictive value (95% CI)	Negative predictive value (95% CI)	Kappa index
MAC-ELISA-HIS^a^	97.4 (92.6-99.5)	98.8 (93.4-100)	99.1 (95.2-100)	96.4 (89.9-99.3)	0.959
MAC-ELISA-mAb^b^	96.6 (91.4-99.1)	91.5 (83.2-96.5)	94.1 (88.3-97.6)	94.9 (87.5-98.6)	0.885

a Hyperimmune serum

b Monoclonal antibody 152

### In-house MAC-ELISA versus IEM systems

A total of 260 paired acuteand convalescent-phase serum samples from suspected chikungunya cases enrolled in one of two ongoing studies of dengue and chikungunya conducted in Managua, Nicaragua (17, 18), were processed by both MAC-ELISA and both IEM systems. All acute samples had been processed previously using the CDC CHIKV rRT-PCR assay, which was used as the gold standard method. Samples that displayed seroconversion to CHIKV-specific IgM or were IgM positive in both of the paired sera were considered positive by the MAC- ELISA. For the IEM, paired serum samples were considered positive when seroconversion or a 4-fold or greater increase of anti-CHIKV antibody titers between the acute and convalescent sera was observed.

Sensitivity, specificity, and positive and negative predictive values for all four serological methods were calculated (Table 3). The four methods yielded a sensitivity of 95.7%, and their specificity ranged from 88.8% (MAC-ELISA-HIS) to 95.9% (IEM-HIS). How-ever, the differences in specificity were not statistically significant. Seven RT-PCR-positive samples were consistently negative by all serological methods. Conversely, 12 pairs of sera were positive by MAC-ELISA-HIS and/or MAC-ELISA-mAb, but negative by rRT-PCR (Table 4). For one of these, CHIKV virus was isolated from the acute sample, which made it a likely false-negative by rRT-PCR (sample #1, Table 4). The remaining 11 samples were considered false positives by one or several of the study’s serological tests. All tested negative for dengue virus infection by MAC-ELISA and IEMdengue (22 – 24).

## DISCUSSION

This study reports the development, standardization, and clinical evaluation of two MAC-ELISAs and two IEMs for the diagnosis and surveillance of chikungunya. In order to evaluate our MAC-ELISA systems, 198 serum samples collected via the national surveillance system were processed using both (MAC-ELISA-HIS and the MAC-ELISA-mAb) ELISA and compared to the CDC-MAC-ELISA as the reference method. A sensitivity of 97.4% and 96.6% and a specificity of 98.8% and 91.5% were obtained for the MAC-ELISA-HIS and MAC-ELISA-mAb, respectively. These results are consistent with results reported for similar assays. For instance, the MAC-ELISA developed by Wasonga and colleagues had a sensitivity of 97.6% and a specificity of 81.3% compared to the CDC-MAC-ELISA. Blacksel and colleagues (26) evaluated a commercial ELISA from Standard Diagnostics, Inc. (Yongin-si, Gyeonggi-do, Republic of Korea), using as reference a method developed by the Research Institute in Medical Sciences of the Armed Forces (Bangkok, Thailand). They obtained a sensitivity and a specificity of 84% and 91%, respectively, in samples collected from patients in the convalescence phase (26). The high sensitivity and specificity of our study might have been due to the high titer of both the HIS and mAbs; in addition, the sera were used at a higher concentration than in the CDC-MAC-ELISA.

A substantial achievement of the present study was to reduce the processing time from 48 hours for the CDC-MAC-ELISA to 2.5 – 3 hours for the in-house MAC-ELISA systems. In addition, the in-house MAC-ELISA assays enabled savings in supplies and reagents because they do not use a non-viral antigen control for each sample. Thus, 90 samples can be processed in one plate without affecting the assay specificity, whereas the CDC technique only allows 45 samples per plate. The decision to use more concentrated samples (1 : 20 instead of 1 : 400 as recommended in the CDC protocol) was made to facilitate the work-flow. The lower dilution allows the samples to be diluted directly into the plate, instead of having to predilute the samples prior to adding them to the plate.

**TABLE 3.** Performance of the in-house serological methods in paired acute and convalescent samples when compared to Real Time reverse transcription polymerase chain reaction designed by Centers for Disease Control and Prevention (CDC rRT-PCR) in acute sample (*n*=260), Nicaragua, 2014-2015

Method	Sensitivity (95% Cl)	Specificity (95% Cl)	Positive predictive value (95% CI)	Negative predictive value (95% CI)	Kappa index
MAC-ELISA-HIS^a^	95.7 (91.3-98.2)	88.8 (80.8-94.3)	93.4 (88.5-96.6)	92.6 (85.3-97.0)	0.851
MAC-ELISA-mAb^b^	95.7 (91.3-98.2)	89.8 (82.0-95.0)	93.9 (89.1-97.1)	92.6 (85.4-97.0)	0.860
IEM-HIS	95.7 (91.3-98.2)	95.9 (89.9-98.9)	97.5 (93.7-99.3)	93.1 (86.2-97.2)	0.910
lEM-mAb	95.7 (91.3-98.2)	94.9 (88.6-98.3)	96.9 (92.8-99.0)	93.1 (86.2-97.2)	0.910

a Hyperimmune serum

b Monoclonal antibody 152.

**TABLE 4.** Analysis of samples positive by MAC-ELISA-HIS^a^ and/or MAC-ELISA-mAb^b ^and negative by Real Time reverse transcription polymerase chain reaction (rRT-PCR) (*n*=12), Nicaragua, 2014-2015

Sample	rRT-PCR	MAC-ELISA-HIS^a^	MAC-ELISA-mAb^b^	IEM-HIS^a^	IEM-mAb^b^
1	Negative	Positive	Positive	Positive	Positive
2	Negative	Positive	Positive	Positive	Positive
3	Negative	Positive	Positive	Positive	Positive
4	Negative	Positive	Positive	Positive	Positive
5	Negative	Positive	Positive	Negative	Negative
6	Negative	Positive	Positive	Negative	Negative
7	Negative	Positive	Positive	Negative	Negative
8	Negative	Positive	Positive	Negative	Negative
9	Negative	Positive	Positive	Negative	Negative
10	Negative	Positive	Negative	Negative	Negative
11	Negative	Positive	Negative	Negative	Negative
12	Negative	Negative	Positive	Negative	Negative

a Hyperimmune serum

b Monoclonal antibody 152.

In addition to the assays for detecting CHIKV-specific IgM antibodies, we developed two Inhibition ELISA methods that measure total anti-CHIKV antibodies. These assays can be used for diagnosis of chikungunya in paired acute and convalescent samples. In addition, they can be used to conduct seroprevalence studies that enable determination of current chikungunya epidemic’s magnitude, and therefore, help predict the potential of future epidemics. A similar method for the detection of total anti-dengue virus antibodies has been used in Nicaragua for 17 years by a clinical study of dengue at the National Pediatric Reference Hospital (Managua, Nicaragua; 18) and for 12 years in the Pediatric Dengue Cohort Study (17). In these studies, the Inhibition ELISA method was used to detect total antibodies in acute and convalescent samples from suspected dengue cases, as well as to detect inapparent dengue virus infections in the Pediatric Dengue Cohort Study using healthy annual samples (27, 28). Based on our experience with this method, two CHIKV IEM were developed using the hyperimmune serum and mAbs (12).

A total of 260 paired acute and convalescent samples collected through both Nicaraguan research studies were processed using all four new serological methods. Results were compared to those from a CHIKV rRT–PCR assay performed on the acute sample. As expected, the sensitivity of the MAC-ELISA was low for samples collected in the acute phase (average of 2.0 days after symptoms onset), but high for samples collected in the convalescent phase (average of 21.6 days after symptom onset). This result was consistent with the fact that detection of IgM is not useful in the first days of illness (1, 2, 4, 8, 26, 29 – 32). The sensitivity of both IEMs was similar to both MAC-ELISAs, reinforcing the utility of these serological methods for diagnosis and surveillance. The specificity of the new serological assays was also consistently high (88.8% – 95.9%). Interestingly, three samples that were negative for CHIKV by rRT-PCR and virus isolation were positive by all four serological methods. This could be due to cross-reactivity with other alphaviruses. However, there is currently no evidence of other alphaviruses circulating in Nicaragua.

### Limitations

One limitation of the study was the lack of a gold standard method for the standardization of the IEM. However, the clinical evaluation of the IEM with the 260 paired samples demonstrated the utility of this technique, with ~95% sensitivity and specificity when compared with testing of acute samples by rRT-PCR. These results are similar to those obtained by Balmaseda and colleagues (33) when the dengue-specific IEM was compared with Hemagglutination inhibition (98.9% sensitivity and 100% specificity). Preliminary results obtained in 87 healthy samples collected several months after an rRT-PCR-confirmed CHIKV infection (average of 103 days after symptom onset) yielded a sensitivity of 92.0% for the IEM-mAb (data not shown). This result confirms the usefulness of this technique for conducting seroprevalence studies in the general population.

Since the standardization and evaluation of these different serological methods was accomplished, the chikungunya MAC-ELISA-HIS assay has been decentralized to 12 regional laboratories in the country, a significant improvement for national epidemiological surveillance. Currently, all of these laboratories are able to identify and differentiate dengue and chikungunya cases using MAC-ELISA. Also, two studies, one local and one national, of chikungunya seroprevalence were conducted using the IEM assays to determine the attack rate of CHIKV infection in Nicaragua (34).

### Conclusions

Two MAC-ELISAs and two IEMs for chikungunya diagnosis and research were developed, standardized, and evaluated by this study. These assays currently support epidemiological surveillance of chikungunya at the national level in Nicaragua, as well as more focused research studies in Managua. Nicaragua has thus become the first country in Central America with the capacity to produce its own reagents for serological diagnosis of chikungunya. Nicaragua’s experience can be extended to other countries, and thus contribute to the sustainability of chikungunya diagnosis and to the fight against the dengue and chikungunya epidemics that are currently affecting the Region of the Americas.

## Acknowledgements.

We are grateful to Michael S. Diamond at Washington University (St. Louis, Missouri, United States) for providing the CHIKV mAbs and 181/25 vaccine strain. We also thank José Luis Soto for his significant contribution in the production of hyperimmune serum; María José Vargas, Stephania Ramírez, and Damaris Collado for their help in processing the samples; Andrea Núñez for her invaluable help in the selection of samples and Douglas Elizondo for his significant help on formatting and editing the text. This work was supported by a supplement to R01 AI099631 (AB) from the United States National Institutes of Health (Bethestha, Maryland, United States).

## Disclaimer.

Authors hold sole responsibility for the views expressed in the manuscript, which may not necessarily reflect the opinion or policy of the *RPSP/ PAJPH* and/or PAHO.
